# 异基因造血干细胞移植治疗T淋巴母细胞白血病/淋巴瘤的疗效及预后因素

**DOI:** 10.3760/cma.j.issn.0253-2727.2023.05.006

**Published:** 2023-05

**Authors:** 澜 罗, 阳 焦, 萍 杨, 艳 李, 文阳 黄, 晓燕 克, 德慧 邹, 红梅 景

**Affiliations:** 1 北京大学第三医院血液科，北京 100191 Department of Hematology, Peking University Third Hospital, Beijing 100191, China; 2 中国医学科学院血液病医院（中国医学科学院血液学研究所），天津 300020 Institute of Hematology and Blood Diseases Hospital, Chinese Academy of Medical Sciences, National Clinical Research Center for Blood Diseases, State Key Laboratory of Experimental Hematology, Tianjin 300020, China

**Keywords:** 早期前体T细胞白血病/淋巴瘤, T淋巴母细胞白血病/淋巴瘤, 造血干细胞移植, 预后, Early T-cell precursor acute lymphoblastic leukemia, T lymphoblastic leukemia / lymphoma, Hematopoietic stem cell transplantation, Prognosis

## Abstract

**目的:**

探讨T淋巴母细胞白血病/淋巴瘤（T-ALL/LBL）采用异基因造血干细胞移植（allo-HSCT）作为巩固治疗的疗效及预后因素。

**方法:**

收集2006年1月至2020年1月北京大学第三医院血液科和中国医学科学院血液病医院收治的119例T-ALL/LBL患者的临床资料。根据巩固治疗方案将患者分为单纯化疗组、化疗序贯异基因造血干细胞移植（allo-HSCT）组、化疗序贯自体造血干细胞移植（auto-HSCT）组，比较各组的5年总生存（OS）率、无进展生存（PFS）率。

**结果:**

有效随访的113例患者中，79例（69.9％）诱导治疗后达完全缓解（CR），17例（15.0％）达部分缓解（PR），治疗总反应率（ORR）达84.9％。诱导治疗获得CR或PR的患者中，化疗序贯allo-HSCT组较单纯化疗组具有更高的5年OS率（55.6％对11.4％，*P*＝0.001）和5年PFS率（54.2％对8.9％，*P*<0.001），而化疗序贯allo-HSCT组与化疗序贯auto-HSCT组比较5年OS率及5年PFS率差异均无统计学意义（*P*＝0.271，*P*＝0.197）。对获得CR的患者进行分析得出同样结论。在仅获得PR患者中，化疗序贯allo-HSCT组仍然较单纯化疗组显示出生存优势（5年OS率分别为37.5％和0，*P*＝0.064）。不同供者来源的allo-HSCT患者5年OS率差异无统计学意义（同胞全相合移植、单倍体移植、无关供者移植分别为61.1％、63.6％和50.0％，*P*>0.05）。早期前体T淋巴细胞白血病（ETP-ALL）患者的诱导化疗缓解率和非ETP-ALL患者比较差异无统计学意义，单纯化疗组中ETP-ALL患者较非ETP-ALL患者5年OS率更低（0对12.6％，*P*＝0.045），而在进行allo-HSCT的患者中，ETP-ALL患者与非ETP-ALL患者5年OS率差异无统计学意义（75.0％对62.9％，*P*＝0.852）。诱导治疗未达CR、巩固治疗未采用移植、LDH≥2倍正常值上限为独立预后不良因素（*P*值均<0.05）。

**结论:**

allo-HSCT可改善T-ALL/LBL患者预后，无论是对诱导治疗达CR还是PR患者，均可作为有效的巩固治疗方法。供者来源不影响allo-HSCT患者长期生存率，allo-HSCT作为巩固治疗手段可以克服ETP-ALL/LBL的不良预后。

## Abstract (en)

**Objective:**

To analyze the efficacy and prognostic factors of allogeneic hematopoietic stem cell transplantation（allo-HSCT）for treating T lymphoblastic leukemia/lymphoma（T-ALL/LBL）.

**Methods:**

This study retrospectively evaluated 119 adolescent and adult patients with T-ALL/LBL from January 2006 to January 2020 at Peking University Third Hospital and Institute of Hematology and Blood Diseases Hospital, Chinese Academy of Medical Sciences. Patients were divided into chemotherapy-only, chemotherapy followed by allo-HSCT, and chemotherapy followed by autologous hematopoietic stem cell transplantation（auto-HSCT）groups according to the consolidation regimen, and the 5-year overall survival（OS）and progression-free survival（PFS）rates of each group were compared.

**Results:**

Among 113 patients with effective follow-up, 96（84.9％）patients achieved overall response（ORR）, with 79（69.9％）having complete response（CR）and 17（15.0％）having partial response（PR）, until July 2022. The analysis of the 96 ORR population revealed that patients without transplantation demonstrated poorer outcomes compared with the allo-HSCT group（5-year OS: 11.4％ *vs* 55.6％, *P*＝0.001; 5-year PFS: 8.9％ *vs* 54.2％, *P*<0.001）. No difference was found in 5-year OS and 5-year PFS between the allo-HSCT and auto-HSCT groups（*P*＝0.271, *P*＝0.197）. The same results were achieved in the CR population. Allo-HSCT got better 5-year OS（37.5％ *vs* 0）for the 17 PR cases（*P*＝0.064）. Different donor sources did not affect 5-year OS, with sibling of 61.1％ *vs* hap-haploidentical of 63.6％ *vs* unrelated donor of 50.0％（*P*>0.05）. No significant difference was found in the treatment response in the early T-cell precursor acute lymphoblastic leukemia/lymphoma（ETP）and non-ETP populations. The ETP group demonstrated lower 5-year OS compared with the non-ETP group in the chemotherapy alone group（0 *vs* 12.6％, *P*＝0.045）, whereas no significant difference was found between the ETP and non-ETP groups in the allo-HSCT group（75.0％ *vs* 62.9％, *P*＝0.852）. Multivariate analysis revealed that high serum lactate dehydrogenase level, without transplantation, and no CR after chemotherapy induction were independently associated with inferior outcomes（*P*<0.05）.

**Conclusion:**

Allo-HSCT could be an effective consolidation therapy for adult and adolescent patients with T-ALL/LBL. Different donor sources did not affect survival. Allo-HSCT may overcome the adverse influence of ETP-ALL/LBL on OS.

T淋巴母细胞淋巴瘤（T-lymphoblastic lymphoma, T-LBL）是一种高度恶性的侵袭性淋巴瘤，约占非霍奇金淋巴瘤的1.7％，好发于儿童及青少年，男性多见。临床上常累及纵隔，表现为前纵隔肿块，骨髓、中枢神经系统累及也较多见，预后差[1]–[2]。急性T淋巴细胞白血病（T-ALL）及T-LBL在细胞形态学及免疫表型方面具有相似性，根据2008年淋巴造血系统疾病WHO分类标准，将T-ALL及T-LBL合并统称为T-ALL/LBL，当骨髓中淋巴母细胞比例<20％诊断为T-LBL，≥20％则为T-ALL。目前此病的治疗方案仍存在争议，T细胞表型淋巴瘤缺乏有效的细胞免疫治疗及靶向药物，强度较大的ALL化疗方案提高了患者的生存率，但在此基础上序贯造血干细胞移植是否能增加患者获益，移植方式、移植供者如何选择等都是亟待解决的问题。基于此，本研究中我们对2006年1月至2020年1月北京大学第三医院血液科和中国医学科学院血液病医院收治的119例T-ALL/LBL进行回顾性病例调查研究，探讨异基因造血干细胞移植（allo-HSCT）对T-ALL/LBL患者的疗效及预后因素，为改善T-ALL/LBL患者的长期生存提供思路。

## 病例与方法

1. 病例资料：本研究纳入2006年1月至2020年1月北京大学第三医院血液科和中国医学科学院血液病医院收治的119例T-ALL/LBL患者，诊断采用2016年WHO淋巴造血系统疾病分类标准[3]。

2. 治疗方案：诱导及巩固治疗中76例患者采用BFM90化疗方案，16例采用Hyper-CVAD A/B方案，余患者采用CHOP样方案，具体参见文献[4]。诱导及巩固治疗后根据患者意愿结合危险度分层选择继续化学药物维持治疗（53例）、自体造血干细胞移植（auto-HSCT，21例）或allo-HSCT（45例）。治疗过程中进行腰椎穿刺送检脑脊液及鞘内注射甲氨蝶呤、地塞米松及阿糖胞苷预防淋巴瘤或白血病细胞对中枢神经系统的侵犯。

3. auto-HSCT：21例auto-HSCT患者中10例预处理方案为含全身照射（TBI）方案：TBI总量10 Gy（肺小于7 Gy），−8、−7、−6 d；环磷酰胺40 mg/kg，−5、−4 d；氟达拉滨30 mg/m^2^，−3、−2、−1 d；阿糖胞苷2 g/m^2^（每日总量，每12 h 1次，静脉输注），−3、−2、−1 d。11例采用BU/CY方案：阿糖胞苷2 g/m^2^（每日总量，每12 h 1次，静脉输注），−10、−9、−8 d；白消安0.8 mg/kg 每6 h 1次，−7、−6、−5 d；环磷酰胺1.8 g/m^2^，−4、−3 d。预处理结束24 h后回输动员采集的自体外周血干细胞。

4. allo-HSCT：45例allo-HSCT患者中同胞全相合移植24例，单倍体移植17例，无关供者移植4例。回输单个核细胞中位数8.33×10^8^/kg，CD34^+^细胞中位数5.8×10^6^/kg。预处理方案：同胞全相合移植：①BU/CY+ATG方案：阿糖胞苷2 g/m^2^（每日总量，每12 h 1次，静脉输注），−10、−9、−8 d；白消安0.8 mg/kg每6 h 1次，−7、−6、−5 d；环磷酰胺1.8 g/m^2^，−4、−3 d；兔抗人胸腺细胞免疫球蛋白（ATG）2.5 mg/m^2^（总量，分4 d输注），−4、−3、−2、−1 d。②含TBI方案：TBI总量10 Gy（肺小于7 Gy），−8、−7、−6 d；环磷酰胺40 mg/kg，−5、−4 d；氟达拉滨30 mg/ m^2^，−3、−2、−1 d；阿糖胞苷2 g/m^2^（每日总量，每12 h 1次，静脉输注），−3、−2、−1 d。亲缘单倍体和无关供者全相合移植：①BU/CY+ATG方案：阿糖胞苷2.5 g/m^2^（每日总量，每12 h 1次，静脉输注），−10、−9、−8 d；白消安0.8 mg/kg每6 h 1次，−7、−6、−5 d；环磷酰胺1.8 g/m^2^，−4、−3 d；ATG 7.5 mg/m^2^（总量，分4 d输注），−4、−3、−2、−1 d。②含TBI方案：TBI总量10 Gy（肺小于7 Gy），−8、−7、−6 d；环磷酰胺40 mg/kg，−5、−4 d；氟达拉滨30 mg/m^2^，−3、−2、−1 d；阿糖胞苷2 g/m^2^（每日总量，每12 h 1次，静脉输注），−3、−2、−1 d；ATG 2.5 mg/kg，−4、−3、−2、−1 d。GVHD预防采用环孢素A（CsA）联合吗替麦考酚酯（MMF）及短程甲氨蝶呤（MTX）。

5. 造血功能重建指标：外周血中性粒细胞绝对计数（ANC）>0.5×10^9^/L持续3 d，未输注血小板情况下PLT>20×10^9^/L持续7 d为造血功能重建。

6. 疗效判定标准：根据2016年NCCN指南推荐的LUGANO淋巴瘤疗效标准，完全缓解（CR）定义为淋巴结、融合团块恢复正常，淋巴瘤相关实验室检查正常，治疗后PET-CT Deauville评分1～3分伴或不伴残留病灶，同时若有骨髓累及，则骨髓象原始幼稚淋巴细胞比例<5％。部分缓解（PR）定义为肿瘤最长径及其垂直线最长径之和（SPD）减少≥50％，治疗后PET-CT Deauville评分4～5分且较基线水平摄取减少，无新发或进展病灶，骨髓原始细胞较原来减少50％以上。疾病进展（PD）定义为出现新病灶或旧病灶最长横径与垂直于最长横径短径之积（PPD）增加≥50％，PET-CT Deauville评分4～5分且较基线水平摄取增高或出现新的FDG高摄取灶，骨髓原始细胞>20％，或较原来减少未达到50％；疾病稳定（SD）定义为除CR、PR、PD以外的疾病状态。总生存（OS）期指从确诊至死亡或随访截止的时间。无进展生存（PFS）期指从确诊至复发或非复发因素导致死亡的时间。

7. 随访：采用查阅患者住院或门诊病历、电话的方式随访。随访截止日期为2022年7月，中位随访时间60（11～154）个月，其中6例失访。

8. 统计学处理：采用SPSS 22.0统计分析软件，计量资料采用*t*检验，计数资料采用卡方检验，生存分析采用Kaplan-Meier法，各组生存率比较采用Log-rank检验，单因素及多因素预后风险因素分析采用Cox回归模型，*P*<0.05为差异有统计学意义。

## 结果

1. 临床特征分析：119例T-ALL/LBL患者中男78例，女41例，男女比例1.9∶1，中位发病年龄27（15～77）岁。114例患者疾病分期为Ⅲ/Ⅳ期，86例患者累及骨髓，其中46例骨髓原始幼稚淋巴细胞比例>20％，诊断为T-ALL，9例（7.5％）诊断为早期前体T淋巴细胞白血病（ETP-ALL）。119例患者中有6例因治疗过程中断或转外院无化疗疗效资料，余113例患者中，79例（69.9％）诱导治疗后达CR，17例（15.0％）达PR，17例患者疾病稳定或进展，均在1年内死亡，治疗总反应率（ORR，CR率+PR率）达84.9％。根据巩固治疗方案将患者分为三组：单纯化疗组53例，化疗序贯allo-HSCT组45例，化疗序贯auto-HSCT组21例。化疗序贯allo-HSCT组中同胞相合供者24例，单倍体供者17例，HLA全相合无关供者4例。三组患者的临床特征详见表1。除年龄及化疗后CR率外，其余临床特征包括性别、临床分期、IPI评分、骨髓受累情况、血清LDH水平、WBC三组间差异均无统计学意义。单纯化疗组年龄较移植组偏大，allo-HSCT组年龄较auto-HSCT组偏大。移植组患者较单纯化疗组诱导化疗CR率更高，但allo-HSCT组和auto-HSCT组比较，差异无统计学意义（*P*>0.05）。

**表1:** 119例T淋巴母细胞白血病/淋巴瘤患者一般临床资料

临床特征	单纯化疗组（53例）	化疗序贯allo-HSCT组（45例）	化疗序贯auto-HSCT组（21例）	*P*值
年龄[岁，*M*（范围）]	28(15~77)	28(17~51)	23(15~34)	0.001
性别[例（%）]				0.993
男	35(66.0)	29(64.4)	14(66.7)	
女	18（34.0）	16（35.6）	7（33.3）	
Ann Arbor分期[例（%）]				0.177
Ⅰ/Ⅱ	3(5.7)	0（0）	0（0）	
Ⅲ/Ⅳ	49(92.5)	44(97.8)	21(100)	
缺失	1(1.8)	1(2.2)	0（0）	
IPI评分[例（%）]				0.577
0~2	28(52.8)	28 (62.2)	16(76.2)	
3~4	16(30.2)	12(26.7)	4(19.0)	
缺失	9(17.0)	5(11.1)	1(4.8)	
骨髓原始细胞[例（%）]				0.594
无	15(28.3)	9(20.0)	4(19.1)	
≥20%	12(22.6)	16(35.6)	8(38.1)	
<20%	23(43.4)	19(42.2)	8(38.1)	
缺失	3(5.7)	1(2.2)	1(4.8)	
血清LDH水平[例（%）]				0.329
≥2倍正常值上限	8(15.1)	3(6.7)	2(9.5)	
<2倍正常值上限	37(69.8)	39(86.6)	18(85.7)	
缺失	8(15.1)	3(6.7)	1(4.8)	
WBC[例（%）]				0.337
>10 × 10^9^/L	10(18.9)	12(26.7)	1(4.8)	
≤10 × 10^9^/L	31(58.5)	31(68.9)	13(61.9)	
缺失	12(22.6)	2(4.4)	7(33.3)	
诱导治疗反应[例（%）]				0.001
CR	23(43.4)	35(77.8)	21(100)	
PR	9(17.0)	8(17.8)	0（0）	
PD/SD	16(30.2)	1(2.2)	0（0）	
缺失	5(9.4)	1(2.2)	0（0）	
ETP-ALL/LBL[例（%）]				0.517
是	5(9.4)	4(8.9)	0（0）	
否	45(84.9)	37(82.2)	21(100)	
缺失	3(5.7)	4(8.9)	0（0）	

**注** allo-HSCT：异基因造血干细胞移植；auto-HSCT：自体造血干细胞移植；Ann Arbor分期：国际标准淋巴瘤分期；IPI：国际预后指数；CR：完全缓解；PR：部分缓解；PD/SD：疾病进展/疾病稳定；ETP-ALL/LBL：早期前体T淋巴细胞白血病/淋巴瘤

2. 疗效及生存分析：我们比较了目前临床中主要采用的两种诱导化疗方案BFM90（76例）及hyperCVAD（16例）对生存的影响，发现两种诱导化疗方案组ORR差异无统计学意义（84.2％对93.8％，*P*＝0.358）。对单纯化疗组、allo-HSCT组和auto-HSCT组进行组内分析，各组中两种诱导化疗方案患者的5年OS率差异均无统计学意义意义（*P*值均>0.05）。

对96例获治疗反应（CR+PR）患者进行生存分析，单纯化疗组5年OS率为11.4％，5年PFS率为8.9％。化疗序贯allo-HSCT组5年OS率为55.6％，5年PFS率为54.2％。化疗序贯auto-HSCT组5年OS率为36.8％，5年PFS率为31.6％。无论allo-HSCT组还是auto-HSCT组均较单纯化疗组具有更高的5年OS率及5年PFS率（OS: *P*＝0.001，*P*＝0.015；PFS: *P*<0.001，*P*＝0.009），而allo-HSCT组和auto-HSCT组比较差异均无统计学意义（*P*＝0.271，*P*＝0.197）（图1）。

**图1:**
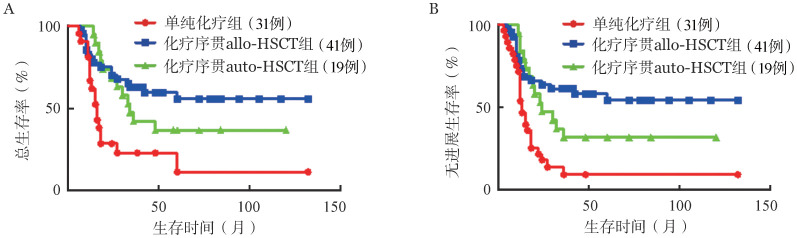
诱导治疗获得反应的T淋巴母细胞白血病/淋巴瘤患者中不同巩固治疗组的总生存（A）和无进展生存（B）曲线

患者基本临床特征分析提示移植组较单纯化疗组具有更高的CR率，为更准确地研究不同巩固治疗方法对预后的影响，我们对诱导治疗后达CR的患者进行分析，发现单纯化疗组5年OS率、5年PFS率分别为25.0％和14.0％，化疗序贯allo-HSCT组分别为59.5％和57.7％，化疗序贯auto-HSCT组分别为36.8％和31.6％。allo-HSCT组较单纯化疗组具有更高的5年OS率及5年PFS率（*P*＝0.011，*P*＝0.001），而allo-HSCT组与auto-HSCT组比较、auto-HSCT组与单纯化疗组比较差异均无统计学意义（*P*值均>0.05）（图2）。

**图2:**
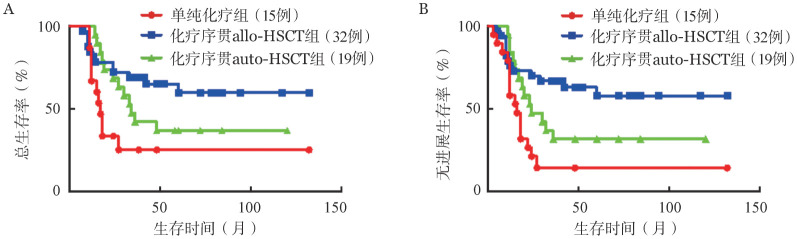
诱导治疗获得完全缓解的T淋巴母细胞白血病/淋巴瘤患者中不同巩固治疗组的总生存（A）和无进展生存（B）曲线

此外，我们分析不同供者来源，即同胞相合供者、单倍体供者及HLA全相合无关供者对allo-HSCT预后的影响，结果显示三组的5年OS率分别为61.1％、63.6％、50.0％，差异无统计学意义（*P*>0.05）。

本研究中接受allo-HSCT及auto-HSCT的所有患者造血功能均顺利重建，allo-HSCT组5年累积复发率为28.2％，auto-HSCT组为68.4％（*P*＝0.009）。接受allo-HSCT的患者中发生移植物抗宿主病（GVHD）11例，肺部感染7例，造血干细胞移植后淋巴细胞增殖性疾病（PTLD）1例。14例患者死亡，其中8例死于移植并发症，6例死于肿瘤复发。21例auto-HSCT患者均未出现移植相关并发症，12例死于肿瘤复发。

本研究中9例（7.5％）患者诊断为ETP-ALL，非ETP-ALL患者和ETP-ALL患者CR率分别为66.0％（68/103）和77.8％（7/9），差异无统计学意义（*P*＝0.715）。9例ETP-ALL患者中，4例以allo-HSCT作为巩固治疗，5例仅进行化疗。未行移植患者中ETP-ALL组患者1年内全部死亡，非ETP-ALL组患者5年OS率为12.6％，ETP-ALL较非ETP-ALL显示出更差的生存（*P*＝0.045）。而在进行allo-HSCT的患者中，ETP-ALL组5年OS率为75.0％，非ETP-ALL组为62.9％，差异无统计学意义（*P*＝0.852）。

3. 预后影响因素分析：在单因素预后分析中，纳入年龄、性别、是否存在纵隔大包块、B症状、IPI评分、Ann Arbor分期、骨髓累及、诱导治疗后是否获得CR、初诊时LDH≥2倍正常值上限、IPI评分≥3分、初诊WBC水平、是否进行造血干细胞移植、是否为ETP-ALL/LBL分型。分析发现初诊时LDH≥2倍正常值上限、IPI评分≥3分、诱导治疗未达CR及巩固治疗未采用移植和预后不良相关（*P*值均<0.05）。将以上因素纳入多因素Cox回归模型分析提示诱导治疗未达CR、巩固治疗未采用移植、LDH≥2倍正常值上限为独立预后不良因素（表2）。

**表2:** T淋巴母细胞白血病/淋巴瘤患者预后的多因素Cox回归分析

变量	*HR*（95% *CI*）	*P*值
接受造血干细胞移植	0.342（0.176~0.663）	0.004
诱导治疗后获得CR	0.446（0.230~0.865）	0.009
LDH≥2倍正常值上限	3.131（1.393~7.037）	0.004

**注** CR：完全缓解；LDH：乳酸脱氢酶

## 讨论

T-ALL/LBL是一种高度恶性的侵袭性血液系统肿瘤，发病机制不明，预后差，目前尚缺乏统一的标准一线治疗方案。ALL儿童化疗方案的应用显著提高了患者治疗缓解率及生存率，然而复发率高仍然是T-ALL/LBL治疗中的难题。研究显示T-LBL治疗后复发率高达50％，长期生存率仅为14％～50％ [5]–[7]。auto-HSCT及allo-HSCT均被应用于T-LBL患者诱导治疗缓解后的巩固治疗中[8]–[10]。早期造血干细胞移植仅用于高危或复发T-LBL患者[11]，但一些研究表明初治达CR的患者接受造血干细胞移植作为巩固治疗仍可获益[12]–[14]。移植方式选择、移植时机及受益人群仍存在争议。一项多中心前瞻性研究对比auto-HSCT及常规巩固化疗的疗效表明两者2年OS率差异无统计学意义（56％对45％，*P*＝0.71）[15]，另一项回顾性临床研究表明化疗序贯auto-HSCT较单纯化疗维持治疗显示出更好的疾病无进展生存[16]。来自浙江大学医学院团队的T-LBL回顾性研究表明在采用儿童ALL化疗方案获得治疗反应的患者中，序贯allo-HSCT较单纯化疗3年OS率更高（72.8％对17.5％，*P*＝0.008）[17]。一项关于allo-HSCT及auto-HSCT在治疗LBL（包括T-LBL及B-LBL）的回顾性对比研究表明，allo-HSCT复发率低但移植相关死亡率高，远期生存两组差异无统计学意义[18]。本研究对96例CR+PR患者进行分析发现无论allo-HSCT还是auto-HSCT均较单纯化疗组具有更高的5年OS率及PFS率，而前两者比较差异均无统计学意义。分析三组基本临床特征时发现移植组较单纯化疗组具有更高的CR率，而诱导缓解是否达到CR为重要的预后相关因素，考虑到移植组更好的远期生存可能为CR率高这一因素所致，因此我们对诱导化疗后达CR部分患者进行统计学分析，获得同样结论。此外，本研究对17例PR患者分析发现，allo-HSCT组仍然较单纯化疗组显示出生存优势（*P*＝0.064），差异无统计学意义考虑与样本量小相关，提示诱导缓解仅达PR患者行allo-HSCT仍可获益。

本研究结果提示，allo-HSCT组较auto-HSCT组具有更低的5年累积复发率，一项来自国内对41例T-LBL患者进行auto-HSCT的研究表明IPI评分中高危组患者移植后3年累积复发率高于中低危组[19]，本研究中IPI中高危组患者移植方式均采用allo-HSCT，我们认为中高危患者进行allo-HSCT可能较auto-HSCT是更好的选择。

本研究还发现不同供者来源的allo-HSCT患者，即供者为同胞相合、单倍体或HLA全相合无关供者时，5年OS率及移植后复发率差异均无统计学意义。鉴于目前存在同胞相合供者的概率较低，无关供者配型成功时间过长，而单倍体供者相对容易找到，因此本研究认为单倍体供者可以作为T-ALL/LBL患者进行allo-HSCT的主要供者来源之一。

ETP-ALL/LBL作为T-ALL/LBL中的高危亚型预后极差，一项来自MD Anderson肿瘤中心关于ETP-ALL/LBL的研究显示ETP-ALL/LBL较非ETP-ALL/LBL患者具有更低的CR率（73％对91％）及更差的生存（中位生存时间不到20个月）[20]。本研究对9例ETP-ALL/LBL患者分析发现，ETP-ALL/LBL患者和非ETP-ALL/LBL患者比较诱导化疗CR率差异无统计学意义。预后分析提示在单纯化疗的患者中ETP-ALL/LBL组较非ETP-ALL/LBL组具有更低的5年OS率，而在进行allo-HSCT的患者中，ETP-ALL/LBL组与非ETP-ALL/LBL组5年OS率差异无统计学意义，因此我们认为allo-HSCT作为巩固治疗手段可以克服ETP-ALL/LBL的不良预后。本研究样本量小，确切的结论需大样本研究证实。

既往对T-ALL/LBL预后影响因素的分析中，>40岁、高LDH水平、IPI评分高、高白细胞水平、Ki-67高表达和骨髓受累等作为潜在危险因素均有报道，但尚未证实T-ALL/LBL成年患者存在确切的危险因素或预后因素[21]–[23]。本研究中，Cox多因素预后分析提示，诱导治疗未达CR、巩固治疗未采用移植及LDH≥2倍正常值上限是影响患者远期生存的独立危险因素（*P*<0.05）。

本研究结果提示对诱导治疗达CR或仅达PR患者，allo-HSCT均可改善T-ALL/LBL患者预后，单倍体供者可以作为主要供者来源之一，allo-HSCT作为巩固治疗手段可以克服ETP-ALL/LBL的不良预后。
